# Unmet needs related to the quality of life of advanced cancer patients in Korea: a qualitative study

**DOI:** 10.1186/s12904-021-00749-8

**Published:** 2021-04-13

**Authors:** Jeehee Pyo, Minsu Ock, Mina Lee, Juhee Kim, Jaekyung Cheon, Juhee Cho, Jung Hye Kwon, Hyeyeoung Kim, Hyeon-Su Im, Young Joo Min, Su-Jin Koh

**Affiliations:** 1 Department of Preventive Medicine, Ulsan University Hospital, University of Ulsan College of Medicine, 877 Bangeojinsunhwando-ro, Dong-gu, Ulsan, 44033 Republic of Korea; 2 Department of Preventive Medicine, Asan Medical Institute of Convergence Science and Technology, Asan Medical Center, University of Ulsan College of Medicine, Seoul, Republic of Korea; 3 Department of Hematology and Oncology, Ulsan University Hospital, University of Ulsan College of Medicine, 877 Bangeojinsunhwando-ro, Dong-gu, Ulsan, 44033 Republic of Korea; 4 Samsung Comprehensive Cancer Center, Samsung Medical Center, Seoul, Republic of Korea; 5 Division of Hematology and Oncology, Department of Internal Medicine, Chungnam National University Sejong Hospital, Chungnam National University College of Medicine, Chungnam, Republic of Korea

**Keywords:** Health services needs and demand, Health-related quality of life, Patient satisfaction, Qualitative research

## Abstract

**Background:**

It has recently been emphasized that the unmet needs of cancer patients should be evaluated more holistically, for example, by exploring caregivers’ perspectives and cross cultural differences. This study explored additional domains or items of unmet needs among Korean cancer patients in reference to the Sheffield Profile for Assessment and Referral to Care (SPARC).

**Methods:**

We conducted four focus group discussions (FGDs) with 15 cancer patients, following a semi-structured format to elicit participants’ health perceptions, comments on SPARC, and opinions on the roles of medical professionals to improve the health-related quality of life of cancer patients. We analyzed the verbatim transcripts using a content analysis method.

**Results:**

The following themes were derived: living as a cancer patient, striving to overcome cancer, changing attitudes toward life after the cancer diagnosis, and ways to live a better life as a cancer patient. The participants asserted the significance of providing adequate treatment information that is easily understood by cancer patients during the conversation between patients and medical professionals. Besides the physical symptoms identified by SPARC, the participants struggled with numbness in their hands and feet and hair loss. Korean cancer patients prominently wished to avoid burdening their family or others in their daily life. They considered the improvement of health behaviors, such as diet and exercise, as part of the treatment, which was not limited to drugs. Furthermore, it was essential to evaluate the value of cancer patients’ lives, as they desired to be helpful members of their families and society.

**Conclusions:**

This study identified additional domains and items of unmet needs of Korean cancer patients and broadened the understanding of unmet needs among cancer patients.

**Supplementary Information:**

The online version contains supplementary material available at 10.1186/s12904-021-00749-8.

## Background

The increasing cancer incidence and prevalence have generated substantial strain not only in Korea but also worldwide [1, 2]. Thus, it is imperative to take measures to reduce the physical, mental, and economic burden of cancer [3–5]. The first step to improve the health-related quality of life of cancer patients and lower the cancer mortality rate involves assessing the unmet needs of cancer patients [6]. Such an assessment not only is necessary to satisfy the unmet needs of cancer patients in general but could also be beneficial to establish strategies to better identify Korean cancer patients’ unmet needs.

Various tools have been developed and applied to assess the unmet needs of cancer patients [7–9]. It has recently been emphasized that unmet needs should be evaluated more holistically, for example, by exploring caregivers’ perspectives and cross cultural differences [9, 10]. In particular, the majority of assessment tools for the unmet needs of cancer patients were developed in specific cultures, mainly Western. Thus, it is expected that analyzing the current status of unmet needs of cancer patients in Eastern countries, such as Korea, and comparing it with other countries will expand our understanding of unmet needs of cancer patients.

An assessment tool for the needs of cancer patients was previously developed with a rigorous methodology in Korea [11], but its utilization rate has been mediocre. One reason for the low utilization rate of that tool was that social interest in the unmet needs of cancer patients remains low; thus, it is essential to increase social interest by conducting more diverse studies on the current status of such needs and their assessment. Qualitative methodology is considered to be more useful for acquiring an in-depth understanding of the unmet needs of cancer patients than are quantitative methods. Some qualitative studies on the issue have been published in Korea [4, 5, 12]. While these studies have deepened the apprehension of the unmet needs of cancer patients in Korea, it would be practical to establish a framework to compare the domains of such unmet needs between countries in different cultures.

Thus, this study examined the unmet needs of Korean cancer patients using qualitative methodology to identify additional domains or items of unmet needs in reference to the assessment domains of the Sheffield Profile for Assessment and Referral to Care (SPARC) [13], and explored the implications of the findings for the assessment of unmet needs of cancer patients. SPARC was chosen in this study because it consists of a greater variety of domains for measuring unmet needs of cancer patients compared to other assessment tools [9, 14]. Moreover, we were able to identify domains or items that can be overlooked when evaluating unmet needs for cancer patients in Asian countries by utilizing the SPARC widely used in Western countries. This may expand our in-depth understanding of cultural and social differences in the assessment of unmet needs of cancer patients and provide the attention points of cultural and social differences in interpreting the results.

## Methods

The purpose of the study was to examine the unmet needs of Korean cancer patients undergoing chemotherapy and to understand the implications for comprehensively assessing and comparing such needs. Specifically, we conducted focus group discussions (FGDs) with cancer patients to induce dynamism through the interaction between participants, leading to a variety of responses [15]. The specific research methods used for the FGDs followed the Consolidated Criteria for Reporting Qualitative Research (COREQ) [16].

### Research team composition

The research team consisted of a total of seven members: three members in the analysis team and four in the supervision team. The analysis team included one preventive medicine specialist, one preventive medicine researcher, and one nurse with 11 years of clinical experience. Two of the members had written dissertations applying qualitative methodology, such as Consensual Qualitative Research and case study, and lectured on such methods. The other had extensive experience in qualitative research execution. The supervision team for research consisted of three specialists in hematology and a professor dedicated to education at the S Hospital Cancer Center.

### Research participants

Typical case sampling was used to recruit participants, as it involves selecting the most “average” individuals with experience in the topic of interest [17]. The participation selection criteria for the study were cancer patients who were currently undergoing chemotherapy. We selected cancer patients undergoing chemotherapy because we considered them to have diverse unmet needs compared to cancer patients receiving other treatments [11]. The research nurse contacted eligible patients for inclusion in August 2019 at the outpatient of cancer center and gave a written explanation of the purpose and contents of the study. A total of 15 patients voluntarily agreed to participate in the study. The participants were also informed that they could withdraw from the study freely, but none did.

### Focus group discussions

Group interviews, such as FGDs, have the advantage of providing a wide variety of responses because of participant dynamics that could be absent in individual interviews [15]. Conversely, they have the disadvantage of being limited in acquiring vast independent reactions from each participant [15]. Given this limitation, the research team acknowledged that it would be challenging to conduct a single FGD with 15 participants. Thus, four FGDs were conducted: two groups underwent two sessions (seven and eight participants, respectively). Together, the preventive medicine specialist and researcher conducted the 2 h FGD sessions by referring to the developed semi-structured guidelines. All FGDs were recorded for transcription and analysis.

The preliminary semi-structured guidelines for the FGDs were developed through discussions between the analysis team researchers and reviews of precedent literature. Subsequently, two of the supervision team researchers reviewed the guidelines, and the analysis team finalized them. The guidelines for the first round of FGDs included discussions on health awareness, opinions about the Korean version of the SPARC [18], and other topics (Supplementary file). SPARC is an assessment tool to identify the unmet needs of cancer patients, comprising eight domains with a total of 45 items (i.e., communication and information issues, physical symptoms, psychological issues, religious and spiritual issues, independence and activity, family and social issues, treatment issues, personal issues), plus one open-ended question [13]. The guidelines for the second round of FGDs covered understanding the quality of life of cancer patients using pictures, whereby the researchers asked participants to take pictures of things affecting their health-related quality of life before the second FGD, identifying significant domains utilizing health-related quality of life surveys, opinions about the role of medical professionals to improve health-related quality of life, and other topics (Supplementary file).

### Analysis

We applied directed content analysis, which reinterprets or infers the implications derived from the raw data through the pre-established theory or perspective of researchers in advance [19]. Moreover, we used field notes to repeatedly check the implications by recalling the non-verbal behaviors of the participants.

The specific analysis was conducted in four steps. In the first step, researchers A and B of the analysis team repeatedly read Korean verbatim transcripts and deduced meaning units individually, and then compared them and discussed their discrepancies. In the second step, researcher C of the analysis team reviewed and finalized the meaning units produced by researchers A and B. In the third step, researchers A and B categorized similar meaning units that shared similar themes. In the fourth step, researcher C and four researchers of the supervision team reviewed the results based on the framework of categories.

### Evaluation of validity

We attempted to secure validity by applying the four evaluation criteria proposed by Guba and Lincoln (1985) [20, 21]. First, the truth value is a trust establishment of the “truth” of the results. Accordingly, one participant from each group confirmed the congruency of the results and verified the statements of the participants and the findings. Second, applicability refers to the application feasibility of the research data in settings other than the research context. This is achieved by collecting data from the participants until there is no more new experience, that is, data saturation was achieved [4]. In the course of the two sessions of FGDs, the researchers continued the interview until different opinions were no longer suggested. Third, consistency indicates that similar results can be obtained by querying individuals with similar experiences or results; accordingly, we compared the results of the study to the experiences of two patients who met the participation criteria but did not participate in the study to confirm their similarity. Fourth, neutrality means that the study results were influenced by the experience of participants, not the bias, motivations, interests, or perspectives of the researchers. This can be guaranteed if the research method and analytical process are described and written in detail, and the researchers strive to eliminate their biases. We consciously tried to exclude prejudice from the beginning to the end of the study by continuously recognizing and recording pre-understandings and pre-notions.

## Results

### Socio-demographic characteristics

The study participants consisted of 14 females and one male. Their average age was 55 years. Most of the participants suffered from breast cancer, and the most common cancer grade was stage 4. Table 1 shows the details of the socio-demographic characteristics of the participants in each group.

**Table 1:** Socio-demographic characteristics of participants

Group	N	Sex	Age	Disease	Stage
A	1	Female	60	Breast cancer, stomach cancer	III
	2	Male	58	Liver cell carcinoma	IV
	3	Female	67	Breast cancer	IV
	4	Female	49	Breast cancer	III
	5	Female	69	Breast cancer	II
	6	Female	41	Breast cancer	IV
	7	Female	51	Breast cancer	II
B	1	Female	61	Breast cancer	IV
	2	Female	52	Breast cancer	IV
	3	Female	52	Breast cancer	IV
	4	Female	54	Breast cancer	II
	5	Female	49	Breast cancer	II
	6	Female	52	Breast cancer	III
	7	Female	63	Pancreatic cancer	IV
	8	Female	52	Breast cancer	IV

### Analysis results

A total of 463 meaning units were derived from the analysis of verbatim transcripts of the four FGDs. They were categorized into four themes: living as a cancer patient, striving to overcome cancer, changing attitudes toward life after the cancer diagnosis, and ways to live a better life as a cancer patient. The overall analysis results are summarized in Table 2. Below are descriptions of the core contents of each theme and subcategory.

**Table 2:** Content analysis results

Themes	Subcategory
1. Living as a cancer patient	1–1. Experience of mental distress with cancer diagnosis or relapse
	1–2. Decreased functions and difficulties in daily life due to physical symptoms during chemotherapy
	1–3. Want to be treated as a person, not as a cancer patient
	1–4. Recognizing a big life change because of changed roles in family and social life
	1–5. Finding reason and meaning of life and getting emotional support and comfort from family relationships
2. Strive to overcome cancer	2–1. Recognizing the importance of positive mind and try to restore mental well-being
	2–2. Try to perform outside activities, such as walking
	2–3. Try to adjust to a new life by seeking information related to diet and exercise
	2–4. Obtaining health information and psychological support from other patients and families
3. Changing attitudes toward life after the cancer diagnosis	3–1. Thinking about the meaning and various aspects of health
	3–2. Looking back on the past life and try to find the meaning of life
	3–3. Try to share things with others
4. Ways to live a better life as a cancer patient	4–1. Need information about treatment, potential side effects, and health management from medical professionals
	4–2. Desire to receive emotional empathy and hear hopeful stories as well as detailed information about care in a full and honest conversation with medical professionals
	4–3. Want to share information and getting support from other patients

#### Living as a cancer patient

##### Experience of mental distress with cancer diagnosis or relapse

When the participants were diagnosed with cancer or relapse, they experienced mental health challenges, such as panic, shock, sadness, anger, and death ideation. To study participants who lived a normal daily life, they felt that the sudden cancer diagnosis was a brink of death or death sentence. One participant said she could not forget that her husband said, “Please cure my beloved wife,” while she was denying the truth. Most study participants mentioned that they were embarrassed and had difficulty recovering from the shock; they kept asking, “Why me?”

*“I was so shocked. Now, I have no tears left to cry … ” (Group 1 Second Participant)*

*“Before I had cancer, I–lived a normal life. I lived as the life goes on. Once I was diagnosed, I questioned ‘Why me?’ ‘What did I wrong?’ then I cried out. I just bawled my eyes out. I could not figure out why I got cancer and what did I wrong in my life … I literally did not harm anybody in my life … ” (Group 2 Fourth Participant)*

Some of our study participants experienced a relapse. They described that it was more shocking to them when they heard the news while they were receiving chemotherapy trying their best to overcome cancer. Although the participants had thought about death when they were diagnosed with cancer, the relapse presented an unacceptable truth. Patients said that they could not accept the relapse.

*“When I had a relapse, I just wanted to die; I felt like a burden to my family.” Group 1 Frist Participant*

*“After 36 cycles of chemotherapy, I was told that they did not see any sign of cancer in my body and I might be cured … … My doctor told to take a 3-month break and evaluate again. However, after 2 months, cancer came back. Even I could not speak a word … I really thought it was the end of my life.” (Group 2 Second Participant)*

##### Decreased functions and difficulties in daily life due to physical symptoms during chemotherapy

Despite striving to live as healthy persons, not cancer patients, pain was a frequent reminder of what they were going through for most participants. Tossing and turning at night in bed because of pain led to feelings of desperation and difficulties in daily life. They experienced depression and despair during chemotherapy and perceived the procedure as endless. Their body hair fell out, hands and feet were swollen, and mouths were sore. They experienced severe mood swings, memory decline, and waves of fatigue. Most participants stated that they gradually saw themselves as cancer patients and could no longer deny it. As a result of various physical symptoms that appeared during chemotherapy, they suffered from decreased function (e.g., standing, sleeping, eating) and encountered challenges in daily life. This led them to experience grief, loss, depression, frustration, and despair.

*“I can’t stand for a long time because it (cancer) has spread to my back (in addition to lumbar herniated intervertebral disc). I cannot lift heavy things.” (Group 1 Fifth Participant)*

*“I was desperate a lot at home. Because of hair loss, I look like a patient with a puffy face. At the first chemotherapy, it was extremely difficult to swallow anything. I felt something inside of throat” (Group 2 First Participant)*

##### Want to be treated as a person, not as a cancer patient

Accepting oneself as a cancer patient is different from being perceived as one by others. Most of the participants did not want to be seen as cancer patients by others, although they accepted themselves as one. In particular, they suffered from not being understood and wanted to be treated as a person and not as a cancer patient by family and others. One participant deliberately put on makeup for a more vibrant appearance. Another reduced socializing time and started hiking alone. They tried not to display their condition. Cancer was a matter beyond the imagination of those who have not experienced it. Most of the participants endeavored not to burden their family or others. They strived to fulfill their duty in the family as a “housewife” or “head of household.”

*“Only few close friends who visit me frequently know that I have cancer. However, if they do not have any family members who have cancer, they have no idea about chemotherapy and how difficult it is. It is really hard … I tried to be patient and endure … I did not express or tell this even to my family members. They would also have pain if I told them staying home.” (Group 1 Fourth Participant)*

*“I do my best at home so that I do not disturb any one in my family. I am doing everything I can do … I told my husband that I don’t need help yet.” (Group 2 Eighth Participant)*

##### Recognizing a big life change because of changed roles in family and social life

Reminiscing––but at the same time endeavoring to pursue their daily routine––most of the participants stated that much had changed because of cancer. One participant with a profession quit her job, and another with a food business sold her restaurant. Foremost, the change in their family role was perceived as a tremendous life transformation. Despite the attempts of participants to execute their role as a family member, they struggled and felt remorse for their failure and the burden they created. The endless chemotherapy generated financial difficulties due to the cost of treatment. Despite cancer preventing them from performing tasks independently and augmenting hardship, they also stated that they experienced another significant transformation in reflecting on their lives and spending more time with their families.

*“I regret a lot about that I handed over my restaurant to others because I have nothing to do at home … ” (Group 2 First Participant)*

*“My husband used to like to drink and gamble, but after I got sick, he does everything considering me. I feel grateful … Now, after we back home, he washes me then I feel like being a patient.” (Group 2 Fifth Participant)*

##### Finding reason and meaning of life and getting emotional support and comfort from family relationships

“Family” was a source of guilt for most participants, but also a factor motivating them to live. One participant with young children felt heavyhearted for their inability to play together, but she also wanted to be in their lives until they grew old. A participant with adult children wished to witness the children’s independence and marriage. A participant with married children wished to overcome cancer, worrying about their spouse left alone after their death. Some participants were sometimes disappointed about their families’ indifferent attitude, as they seemed to have forgotten that they were cancer patients. Nevertheless, the existence of a “family” was a reason to live and a source of comfort and strength.

*“My husband is the most concern in my mind, because my kids are now all grown- ups.” (Group 2 Eight Participant)*

*“I got sick when my kid was a sophomore of the middle school–age 15. He did not make any troubles at school and think and take care of me when he can. He calls me once a month, and I cry a lot when he asks me about my health and tells me he misses me. I think a lot how I can be healthy again and meet my son again.” (Group 1 Fourth Participant)*

#### Strive to overcome cancer

##### Recognizing the importance of positive mind and try to restore mental well-being

The first attempt to overcome cancer was “accepting cancer.” Most participants recognized the importance of the mental realm; they endeavored to embrace positive thoughts, appreciation, and a bright and joyful mind, and to restore their mental well-being. Some of them experienced a tearful emotional rollercoaster and an unstable state of mind, although they desired to think positively when facing fear. They cherished hope for a small change to accept cancer and live positively. They also tried to obtain comfort through their religious practice and avoid stress.

*“I could not receive surgery because I had bone metastatic. One day, I live with gratitude then another day, I cry … ” (Group 2 Sixth Participant)*

*“I think you can overcome many things through a religious life … Because I have a religion, things like death does not bother me like that gentleman (Sixth Participant) mentioned. Now I do only wish to be cured as soon as possible … ” (Group 1 Seventh Participant)*

##### Try to perform outside activities, such as walking

Many participants discovered their genuine selves, not the life assigned to a role, despite their bodies becoming weak after the cancer diagnosis. One participant started painting, and another began to grow flowers; one who enjoyed traveling pushed their limit by setting the goal of a trip. One participant did not quit their job because it provided a sense of accomplishment. Most of them persistently engaged in mild hiking to improve their health and ventured to identify their inner selves.

*“I kept thinking while I was watching the sunset, ‘I would like to go to somewhere, such as Jeju Island or Jiri Mountain and stay for a week.’ Then I made up my mind to go there in October.” (Group 2 Sixth Participant)*

*“Every day, I walk around a small mountain near my house except one or two days when I really cannot control my body: on the day I have an outpatient visit or on one or two days after I have chemotherapy, I am too exhausted to get out of my bed on those days.” (Group 1 First Participant)*

##### Try to adjust to a new life by seeking information related to diet and exercise

Diet and exercise were elements that the most participants paid careful attention to in order to restore their health. Diet and health were inextricably associated despite the canker sores in the mouth and the difficulty of appetite loss. Several participants obtained information on diet through acquaintances, fellow patients, broadcasting media, and herbal medicine studies. Subsequently, they adjusted their diet after receiving their physician’s advice or constitution fit verification. They also tried to exercise to improve their health.

*“When we are hospitalized, we usually talk a lot among patients. ‘Oh, that was good,’ or ‘It turns out to be something like that.’ So, I often think like ‘Oh, is that so?’” (Group 1 Seventh Participant)*

*“I started to learn about herbs and edible greens from this year. I learned which herbs are good for cancer or good for my body. I tried to cook and eat food using herbs and edible greens.” (Group 1 Fourth Participant)*

##### Obtaining health information and psychological support from other patients and families

Some participants claimed that the support of those around them had been a remarkable help in all processes of accepting and overcoming cancer. After the cancer diagnosis, they started to avoid people; their faces looked dark, and they were hurt by those who spoke lightly about cancer. Nonetheless, one sweet word from family, friends, fellow patients, or religious community members gave them great courage and comfort, and these persons were a source of health information and psychological support.

*“There were many times my friends gave me immense help. It’s quite comforting.” (Group 1 Seventh Participant)*

*“I don’t really talk to other people about myself (cancer). Professors are always busy, and I do not have many friends. I talk mostly to a monk at the temple I usually go and pray. This is how I manage.” (Group 2 Second Participant)*

### Changing attitudes toward life after the cancer diagnosis

#### Thinking about the meaning and various aspects of health

Most of the participants realized that they were ignorant of their health when they reflected on the course of their struggle against cancer. The loss of health caused by cancer made them think about the meaning of health. Most of them defined health as a state in which they could do their desired activities without sickness. They viewed the meaning of health considering multifarious aspects: performing exercise, having a daily routine besides professional work, thinking positively, excluding stress, having fun, being happy, and more.

*“I realized that ‘being healthy’ or ‘optimal health status’ is when you can do what you want whenever you want.” (Group 1 First Participant)*

*“I was ignoring seniors who collected recycles on the street with hunched back, but cancer change my thought on them: they were able to work because they are healthy and it is very thankful situation.” (Group 2 Fifth Participant)*

#### Looking back on the past life and try to find the meaning of life

The cancer diagnosis and endless chemotherapy process led participants to feel as if their lives were judged. They also were compelled to look back on whether they had lived an improper life. This reflection inspired them to live a respectable life, understand others, and feel appreciation for the present moment. Looking back on life and changing their perspective on it, they attempted to do their best at every moment.

*“I had more than 50 cycles of chemotherapy. I really had a hard time. I looked back on my past life and changed my attitudes toward life by recalling things I did wrong in my life. Now I tried to live with better attitudes. I try to behave and try not to turn down others … ” (Group 1 Fourth Participant)*

*“Inside of my mind, there are people bother me, hurt me. Then I tried to think that is nothing … .it really depends on how I think … .I think every individual is the same and try not to keep anything in my mind.” (Group 2 Eight Participant)*

#### Try to share things with others

Some participants who contemplated the idea of health and reflected on life developed a cherishing mind rather than resentment. One participant wished to give up on their life, but they started volunteer work to do good deeds for others. Other participants shared vegetables from their garden to appreciate happiness or gifted self-made paintings to spread warmth.

*“Chemo totally got to me a few years ago. I questioned why I had to live because there was nothing I could do during that time. One day, suddenly, a thought crossed my mind; the idea of doing good deeds for someone. It made me start volunteer.” (Group 1 Fourth Participants)*

*“There are things I can share with others, you know. In fact, we don’t eat much of what we grow in our family garden. To others, like I can do something and help them … ” (Group 2 Eight Participant)*

#### Ways to live a better life as a cancer patient

##### Need information about treatment, potential side effects, and health management from medical professionals

Most of the participants asserted that it was essential to receive information about cancer and treatment to live a better life as a cancer patient. They experienced a sense of floating alone in a vast ocean when they were diagnosed with cancer and had no information on health management. In addition, experiencing unexpected side effects of chemotherapy generated feelings of despair. They wanted detailed information about cancer, lifestyle, and side effects. They were also willing to participate in lectures related to such topics.

*“At first, I had no idea about what doctors told me to do … I mean, it would be helpful for patients to manage things in daily life if they are informed of specific needs. If patients take care of themselves well, they could receive chemotherapy with more comfort and function better during treatment.” (Group 1 First Participant)*

*“It would be helpful if health professionals, such as nurses, provide information about details about self-care and health management during chemotherapy, for example, telling specific ways to do exercise during chemotherapy, patients would live in a better condition and be more active.” (Group 1 Second Participant)*

#### Desire to receive emotional empathy and hear hopeful stories as well as detailed information about care in a full and honest conversation with medical professionals

“I wish the medical professionals would give me hope”––this statement indicated that the participants relied on medical professionals the most. Most of the participants were not satisfied with the disease-related conversations with their attending doctors. With three “yes” replies, the check-up was finished; they were distressed about the attitude of the attending doctors who were unable to empathize with their anxiety. They expected a meticulous explanation of the test results provided. They were content to hear from nurses, not necessarily the attending doctor.

*“I wish to hear more about it in detail–about the disease … I understand that professors have limited time … .but someone else could … I would like to know how detail information about progress of the disease. Time for each visit is too short for hearing everything I want.” (Group 1 Sixth Participant)*

*“I was certainly not pleased with it. I mean, the visit was over in less than 1 minute. I waited for a while to see the doctor … (The doctor said) the results of the blood test are good. let’s do this and do that … that was it … . Can you imagine how I felt? I collapsed.” (Group 2 Fourth Participant)*

#### Want to share information and getting support from other patients

Some participants expressed that it was profoundly comforting and pleasant to share their experiences with several fellow patients through their participation in the study. One participant gained hope after meeting fellow patients and participating in shared conversations. Most of them acknowledged the strength that “conversation” provided them and realized the necessity of meeting fellow patients who experienced and empathized with the same challenges, which provided consolation.

*“When I get to hear stories from various people, I could share what I have and ask how others are dealing with it … I get to hear about those things, and it would be nice to share my stories, like how far my disease has progressed and how I should go about with my treatment. But nothing like that exists … I thought it would be nice to have that.” (Group 1 Sixth Participant)*

*“I think the communication between patients with the same disease is very important … I could tell my situation and ask about how others do; for example, when I told other patients that I am experiencing skin peeling on my hands after chemotherapy, if other patients told me they had the same experience–this would give me a big comfort. It would be great if there is a way patients can talk to each other.” (Group 1 Seventh Participant)*

## Discussion

In the present study, we conducted a total of four FGDs with 15 cancer patients undergoing chemotherapy in Korea to examine their experiences and identify their unmet needs in-depth. Four themes were derived from the FGDs analysis: living as a cancer patient; striving to overcome cancer; changing attitudes toward life after the cancer diagnosis; and ways to live a better life as a cancer patient. Based on the analysis results, we examined whether there were domains or items that had been overlooked or underestimated in terms of the unmet needs of cancer patients. These results may contribute to a more comprehensive assessment of the unmet needs of cancer patients and suggest implications for improving existing tools to assess them. The results would also facilitate an examination of the cultural and sociological differences among cancer patients.

Specifically, Table 3 demonstrates whether there were domains or items to be considered regarding the unmet needs of Korean cancer patients in reference to the domains of SPARC, one of the representative cancer patients needs assessment tools. The first SPARC domain is related to communication and information. Precedent studies have reported a high demand for information from cancer patients [11, 22]. The participants in our study also emphasized that receiving information about cancer and treatment was essential for a better life as a cancer patient. They wished for understandable information from a cancer patient’s point of view rather than formal information. It was also deemed important to be asked if they wanted additional information while the conversation with medical professionals was harmonious [23]. Therefore, in the domain of communication- and information-related issues, items on satisfaction with the level of understanding and the quality of communication as a result of information delivery could be enhanced.

**Table 3:** Additional considerations for assessing unmet needs of cancer patients

Category		Sheffield Profile for Assessment and Referral to care (SPARC)	Additional and revised items
Communication and information issues	Communication within the medical field	1. Have you talked to any of the people below about your condition? Primary care physician/nurse/persons of religion/social worker/family/other (Please write down the specifics for “other”)	· Do you want information about the state of your cancer, treatment method, side effects of your medications, and health care? · Do you want emotional empathy and a message of hope? · Did you understand the information received from the medical professionals correctly? · Would you like to receive more information about your medical or health condition from your medical professionals? · Were you able to freely express your opinion as a patient during the conversation with medical professionals?
	Communication about information delivery		
Physical symptoms		Have you ever struggled or suffered the following problems in the past month? 2. Pain, 3. Memory loss, 4. Headache, 5. Dry mouth, 6. Oral pain, 7. Shortness of breath, 8. Cough, 9. Nausea, 10. Vomiting, 11. Stool problems (constipation, diarrhea, incontinence), 12. Bladder problems, 13. General weakness, 14. Fatigue, 15. Night sleep disorder, 16. Daytime sleepiness, 17. Loss of appetite, 18. Weight change, 19. Difficulty swallowing 20. Worry about appearance change, 21. Anxious and impatient, 22. Symptoms do not seem to improve	· Added symptoms of numbness in hands and feet, edema, hair loss, and loss of memory. · Added items about standing and walking. · Needs a detailed list of symptoms according to the type of carcinoma and chemotherapy.
Mental issues	At the moment of cancer diagnosis	23. Anxiety, 24. Depression, 25. Confusion, 26. Difficult to concentrate, 27. Loneliness, 28. Everything seems difficult, 29. Living seems to be meaningless, 30. The thought of ending everything, 31. My condition affects my sexual life	· Added denial, anger, resentment, sadness. · Experienced trauma. · Support consultation is required for cancer diagnosis.
	Chemotherapy process		· Added frustration and despair. · Need for supportive companion due to the experience of frustration while feeling ambivalent about restoring mental well-being.
Religious and spiritual issues		32. Worrying about death and dying 33. Religious or spiritual needs not being met	· Do you want to receive counseling (or prayer) from a religious person?
Independence and activity		34. Nothing I can do alone (loss of independence) 35. Changes in daily life such as washing face, bathing, and toileting 36. Changes to everyday chores such as cooking and cleaning	· The word “activity” tends to be widely interpreted in a social sense rather than in a personal sense in Korea.
Family and social issues		37. Sense of not being understood about what you want by others 38. Worrying about how your illness affects your family or others 39. Sense of inability to receive help from family or others 40. Need for more help than you can get from family or others	· Revision feedback on Question 37: Sense of unawareness of my health condition by family or others · Revision feedback on Question 38: Sense of burden (or guilt) on the family due to my cancer · Difficulty maintaining a role as a family member
			· Has the area of my activity decreased due to my appearance changes? (Experience of disconnection)
Treatment-related issues	Dietary restriction	41. Treatment side effects 42. Worrying about long-term side effects of treatment	· Request information on health supplement food · Request information on eating habit improvement
	Exercises		· Demand for individualized exercise programs considering the patient’s condition
	Medicine side effects		· Demand for information on side effects and countermeasures for chemotherapy
Personal issues		43. Do you need assistance with personal business? 44. Would you like to speak with another expert about your condition or treatment? 45. Do you want more information about the following? My condition and care providing/treatment/other forms of assistance/financial assistance/other (Please write it down the specifics for the other)	· Need to consider the meaning of life–explore activities with sense of value

The second SPARC domain is that related to physical symptoms. The Korean participants noted that they acknowledged their cancer patient identity only after suffering pain during chemotherapy. As precedent studies emphasized that the resolution of physical symptoms, such as pain and fatigue, was important for cancer patients [3], it would be essential to reduce the decline in cancer patients’ sleep quality and difficulties in daily life. It was also suggested to include additional symptoms, such as numbness and hair loss. Although SPARC asks about 21 types of physical symptoms, the main physical symptoms experienced may differ by the type of cancer and treatment. Realistically, it could be challenging to inquire about all possible physical symptoms in a questionnaire. Therefore, it is imperative to perform screening to select the symptoms that need to be preemptively identified by cancer and treatment type in advance.

The third and fourth SPARC domains are related to mental and religious/spiritual issues, respectively. The participants asserted that psychological support counseling was essential in all stages of cancer, from diagnosis to chemotherapy. Previous studies also argued that it is important to develop a program for the integrated psychological management of cancer patients, as they might continue to experience psychological difficulties after completing chemotherapy [4]. Participants in the study said they felt psychologically supported and gained hope through FGDs with their fellow patients. As such, it is expected that self-help cancer patient groups will compensate for the limitations of psychological support counseling. Moreover, it is important to provide religious support to patients. Although not all participants had religious beliefs, those with the beliefs were comforted by their religious life and overcame their fear of death.

The fifth SPARC domain is that of independence and activeness. Here a distinctive discrepancy in terms of culture and society between East and West was detected. While SPARC concentrates on the ability of cancer patients to lead their daily life independently, Korean participants were more prominent in their willingness not to burden others, such as their family, because of cancer. In Korea, regarding independence and activeness, it is more distressful to be a burden to others than to be unable to perform tasks without help. Therefore, it is anticipated that providing support not only to cancer patients but also to their caregivers would catalyze the reduction of their psychological burden [10, 24].

The sixth SPARC domain comprises family and social issues. The family was a motivating factor for overcoming cancer but was also related to a sense of guilt. It is considered a characteristic of Asian cultures that they prioritize solidarity over independence in family relationships [25, 26]. Above all, for cancer patients with young children, it is crucial to provide resolutions for child care challenges, as they feel the most guilt toward their children [24]. Moreover, an improvement in social perception needs is required; cancer patients received support from acquaintances but also avoided people because of their deteriorated appearance, and they were hurt by people speaking easily of cancer [26]. Furthermore, in the case of female cancer patients, support measures for appearance changes may increase their confidence in social life.

The seventh SPARC domain consists of treatment-related issues. Similar to the domain of communication and information, the participants were particularly interested in the provision of information about the side effects they might experience during the treatment process [27]. They, however, did not limit their notion of treatment to drugs but also considered improving health behaviors, such as diet and exercise. Thus, it is necessary to offer information about ways to enhance eating habits and exercise according to the physical condition of cancer patients as well as assessing their need for information. Since the adoption of health behaviors by cancer patients could prevent the occurrence of other chronic diseases, it is essential to promote health behaviors, such as smoking cessation, abstinence of alcohol, and physical activity enhancement [28]. It is also necessary to develop a strategy for health behavior improvement, as the diagnosis of cancer could be a significant opportunity for health behavior change [29].

The eighth SPARC domain comprises personal issues. In this domain, it is necessary to understand how cancer patients think about the meaning of life. The participants wanted to be helpful for their families and society, even if they had cancer. Thus, it is assumed that returning to work or retaining their profession is not only financially beneficial but also helpful to maintain a normal life and restore self-esteem [30]. Creating a role to aid other cancer patients through self-help groups could also be considered as a strategy to stimulate cancer patients to appreciate the value of life.

The limitation of the present study was the inability to explore the opinions of patients with various types and stages of cancer. We conducted FGDs with patients undergoing chemotherapy, who were expected to have the most unmet needs, and excluded patients in the early stage. Further studies are thus needed to include early-stage cancer patients, as they might also have unmet needs. Moreover, most of the participants were women; in further studies, including more male patients could expand our understanding of the unmet needs of cancer patients. A one-time study on the unmet needs of cancer patients could not comprehensively evaluate the entire scope of unmet needs; therefore, it is necessary to regularly research domains or items that medical professionals may be missing and conduct further studies on related issues.

## Conclusion

This in-depth study examined the unmet needs of Korean cancer patients through four FGDs with 15 cancer patients. Furthermore, we identified additional domains or items of unmet needs of cancer patients by comparing our results with the domains of SPARC, a representative assessment tool for the unmet needs of cancer patients. We also investigated the implications of our findings for improving the assessment of unmet needs in this patient group. In summary, contents, such as the quality of communication with health professionals, feelings of guilt and burden toward the family, support for improving health behaviors, and navigating the meaning of life should also be addressed when assessing the unmet needs of Korean cancer patients. In order to develop and implement policies to alleviate unmet needs for cancer patients, studies should begin with a comprehensive measurement of various unmet needs of the identified cancer patients.

## Supplementary Information

**Additional file 1.**

## Data Availability

The Korean verbatim transcripts are available from the corresponding author on reasonable request.

## Abbreviations

SPARC: Sheffield Profile for Assessment and Referral to Care
FGD: Focus group discussion
