# Supplementary material for: Unmet needs related to the quality of life of advanced cancer patients in Korea: a qualitative study
Source: BMC Palliat Care. 2021 Apr 13;20:58. doi: 10.1186/s12904-021-00749-8 (PMC8045373; doi:10.1186/s12904-021-00749-8)
Supplement: Supplementary file 1 — Additional file 1. [file 12904_2021_749_MOESM1_ESM.docx]

**FGD Guideline for the Research of Unmet Needs Related to the Quality of Life of Advanced Cancer Patients in Korea**

Version 1.0

*** Primary FGD**

| Ice-breaking (10-minute) |
| --- |

1. FGD Introduction

⦁ Introduction and explanation of the discussion purpose

⦁ Introduction of the discussion process

- Notify the FGD recording and confirm approval of the recording from participants

- Inform of the discussion process and precautions

- Inform of personal information protection

2. Participation Introduction

⦁ Self-introduction of a participant’s socio-demographic characteristics: Gender, age, occupation, education level, medical history, and more

| Perception of health and health-related quality of life (30-minute) |
| --- |

3. Perception of Health

1) “What does it mean to be healthy?”

▶ Inquiry Questions

- What are the characteristics of being healthy? How would you describe a healthy person?

- Would you consider yourself healthy? On what basis would you consider yourself healthy? On what basis would you consider yourself not healthy?

- What is the difference between a healthy person and an unhealthy person?

2) “What does it mean to be unhealthy?”

▶ Inquiry Questions

- Investigate health-related history and identify the meaning of being unhealthy

- Have you ever experienced poor health? Could you explain what it was like at that time? Could you think of someone who is or was unhealthy? What makes you consider them unhealthy?

3) What are the problems that would most significantly impact your quality of life?

▶ Inquiry Questions

- What is the most frightening health problem to you? Why?

- Which one has the most impact on your quality of life? Why?

| Perception of the established SPARC_Korean as an assessment tool (45-minute) |
| --- |

4. Survey using SPARC_Korean

⦁ Brief introduction of the survey

⦁ Filling out the questionnaire

5. Understanding of SPARC_Korean

⦁ What did you think of the questionnaire overall?

⦁ Did you find the questionnaire challenging to answer? What difficulties did you have?

▶ Inquiry Questions

- Shall we compare the answers from the questionnaire? What are the answers? What are the reasons for your answers?

- Do you think the questionnaire could adequately identify health-related problems or challenges of your acquaintances?

- Which domains of the questionnaire will be important if you explain your problems? Why? Which domains would be not important? Why?

- What do you think of the format and the use of vocabulary of the response option? Does it need any modification?

- On what basis did you score the overall pain? How would the score increase by one-point? What changes would increase the score by one-point? What changes would decrease the score by one-point?

- What are the advantages and disadvantages of the questionnaire overall? What measures should be taken to improve the questionnaire?

| Additional discussion (5-minute) |
| --- |

6. Discussion conclusion

⦁ Inquiring additional question: Are there any additional comments or questions?

⦁ Discussion conclusion

- Express gratitude for the participation

- Announce and give assignments for the next discussion: take pictures of elements that most affect the quality of their lives, the parts that reflect the quality of their lives, and the parts necessary to improve the quality of their lives and more (Taking multiple pictures are also permitted)

- Explain the photo transferring procedure

*** Secondary FGI**

| Ice-breaking (10-minute) |
| --- |

1. FGD Introduction

⦁ Introduction and announcement of the discussion process

- Notify the FGD recording and confirm approval of the recording from participants

- Inform of the discussion process and precautions

- Inform of personal information protection

2. Review of last FGD

⦁ Take turns listening to each participant’s recollections and impressions of the last discussion

⦁ Chat about their previous week

⦁ Review their performances for the last FGD

| Photovoice (40-minute) |
| --- |

3. Identify the quality of life of advanced cancer patients via photovoice

⦁ “Let’s continue the discussion with the pictures that each of you brought today.”

- Present the pictures on a screen

▶ Inquiry questions

- What is the meaning of this picture?

- What impressions do all of you receive from these pictures?

- What systematic modification would improve the quality of life need?

| The significant domain of the health-related quality of life (20-minute) |
| --- |

4. Survey on the health-related quality of life

⦁ Brief introduction of the questionnaire

⦁ Filling out the questionnaire

⦁ What did you think of the questionnaire overall?

⦁ Did you find the questionnaire challenging to answer? What difficulties did you have?

▶ Inquiry questions

- Shall we compare the answers from the questionnaire? What are the answers? What are the reasons for your answers?

- Which domains of the questionnaire will be important if you explain your problems? Why? Which domains would be not important? Why?

5. Five problems that would be considered the most influential health-related problems

▶ Inquiry questions

- Between the five problems, what is the most significant health-related problem? Why?

- Which one has the most impact on your quality of life? Why?

| Role of medical professionals and others on the improvement of health-related quality of life (15-minute) |
| --- |

6. Role of medical professionals on the improvement of health-related quality of life

⦁ What assistance could medical professionals offer on the improvement of health-related quality of life?

- Investigation on the domains that can fulfill the unmet needs

▶ Additional related question

- Other than the medical professionals, could anyone else perform a significant role in improving of the health-related quality of life?

| Additional discussion (5-minute) |
| --- |

7. Discussion conclusion

⦁ Inquiring additional question: Are there any additional comments or questions?

⦁ Discussion conclusion

- Express gratitude for the participation

- Explain the possible additional inquiry via phone

- Inform the future plan for the transcription content confirmation and analysis review
